# Evaluation of the informatician perspective: determining types of research papers preferred by clinicians

**DOI:** 10.1186/s12911-017-0463-z

**Published:** 2017-07-05

**Authors:** Boshu Ru, Xiaoyan Wang, Lixia Yao

**Affiliations:** 1 Department of Software and Information Systems, The University of North Carolina at Charlotte, Charlotte, NC 28223 USA; 2 Department of Family Medicine and Center for Quantitative Medicine, The University of Connecticut Health Center, Farmington, CT 06030 USA; 3 Department of Health Sciences Research, Mayo Clinic, Rochester, MN 55905 USA

**Keywords:** Clinicians’ reading preference, Medical subject headings, Literature recommender systems

## Abstract

**Background:**

To deliver evidence-based medicine, clinicians often reference resources that are useful to their respective medical practices. Owing to their busy schedules, however, clinicians typically find it challenging to locate these relevant resources out of the rapidly growing number of journals and articles currently being published. The literature-recommender system may provide a possible solution to this issue if the individual needs of clinicians can be identified and applied.

**Methods:**

We thus collected from the CiteULike website a sample of 96 clinicians and 6,221 scientific articles that they read. We examined the journal distributions, publication types, reading times, and geographic locations. We then compared the distributions of MeSH terms associated with these articles with those of randomly sampled MEDLINE articles using two-sample Z-test and multiple comparison correction, in order to identify the important topics relevant to clinicians.

**Results:**

We determined that the sampled clinicians followed the latest literature in a timely manner and read papers that are considered landmarks in medical research history. They preferred to read scientific discoveries from human experiments instead of molecular-, cellular- or animal-model-based experiments. Furthermore, the country of publication may impact reading preferences, particularly for clinicians from Egypt, India, Norway, Senegal, and South Africa.

**Conclusion:**

These findings provide useful guidance for developing personalized literature-recommender systems for clinicians.

**Electronic supplementary material:**

The online version of this article (doi:10.1186/s12911-017-0463-z) contains supplementary material, which is available to authorized users.

## Background

Medicine is a field that is continually changing as knowledge of disease and health continues to advance. In the age of translational medicine, clinicians constantly face challenges in transforming scientific evidence into ordinary clinical practice. Peer-reviewed scientific literature is a major, minimally biased resource for scientists and other researchers to communicate their discoveries based on experiments and their findings from rigorously implemented trials and thoughtfully balanced clinical guidelines. Thus, remaining current on medical literature can help clinicians provide the best evidence-based care for their patients [1].

Nevertheless, a widely held belief is that most clinicians rarely read scientific literature on account of their hectic schedules evaluating patients and preparing the required paperwork. According to a survey by Saint et al. US internists reported that they read medical journals for an average of 4.4 h per week in 2000 [2]. Analysis of the web log of 55,000 Australian clinicians by Westbrook et al. revealed an average of 2.32 online literature accesses per clinician per month in a period from October 2000 to February 2001 [3]. Moreover, a 2007 survey report by Tenopir et al. reported that 666 pediatrician participants spent 49 to 61 h per year (equivalent to 0.94 to 1.2 h per week) reading journal articles [4]. In the same year, McKibbon and colleagues showed that primary care physicians not affiliated with an academic medical center in the US accessed an average of one online journal article per month, while specialists not affiliated with an academic medical center accessed an average of 1.9 online journal articles each month [5].

The time constraint of clinicians is the most commonly suggested reason for the small number of articles read. Moreover, access to journals and publication databases is sometimes limited to clinicians with academic affiliations, which can subsequently limit the number of articles accessed and read. Another consideration is whether scientific journal reading comprised everyday practice during training. The majority of clinicians presently in practice were trained in the pre-digital era and likely did not learn the literature-searching skills necessary to keep updated [6–9]. Furthermore, they may not have the advanced technical knowledge (e.g., statistical modeling) often required to understand and apply the findings in scientific articles to clinical practice [7, 10, 11].

Moreover, the aims of clinicians by reading are often discordant with the goals of researchers in their publishing endeavors. A clinician usually seeks information that is relevant to his/her respective medical practice, whereas many researchers do not emphasize the clinical relevance of their work to an extent that is sufficient to address the clinician’s needs [8, 10, 12]. Researchers often use specialized and nuanced language to describe complex medical discoveries in scientific literature. Such language can be opaque for practicing clinicians. In addition, clinicians may be frustrated by many discrepancies and knowledge gaps in the literature [13–16] because they require conclusive and actionable information in practice.

To address this issue, some researchers have strived to improve the user experience with literature search engines. They have added the functions of sorting and clustering search results, as well as extracting and displaying semantic relations among concepts [17]. In addition, considerable research efforts have been made in building intelligent recommender systems that automatically recommend literature to users by employing content-, collaborative-, or graph-based filtering methods [18–22]. Although these studies differed in the methods used, they all targeted scientists and other researchers for whom literature review is an integral part of their daily work.

Clinicians, on the other hand, who search for and read scientific literature, are transcending their daily routine to satisfy their intellectual curiosity, increase their awareness of the latest scientific advancements, and obtain knowledge for treating their patients. The distinction in this regard between the needs of clinicians versus professional researchers has inspired us to investigate the specific needs of clinicians and their preferences for reading literature. Our goal is to learn from clinicians what types of medical research papers they prefer to read and their modes of accessing and assimilating the knowledge.

In this study, we identified a group of clinicians and the scientific papers they were likely to read from the CiteULike.org website. We investigated what type of job they perform, in what specialty they practice, in which country they live and work, the length of time they have practiced, and what scientific research was interesting to them. This type of systematic examination of clinician reading libraries is an important pre-market evaluation for developing a personalized literature-recommender system to improve clinician reading experiences and overall promote the reading of scientific literature.

## Methods

**Fig. 1:**
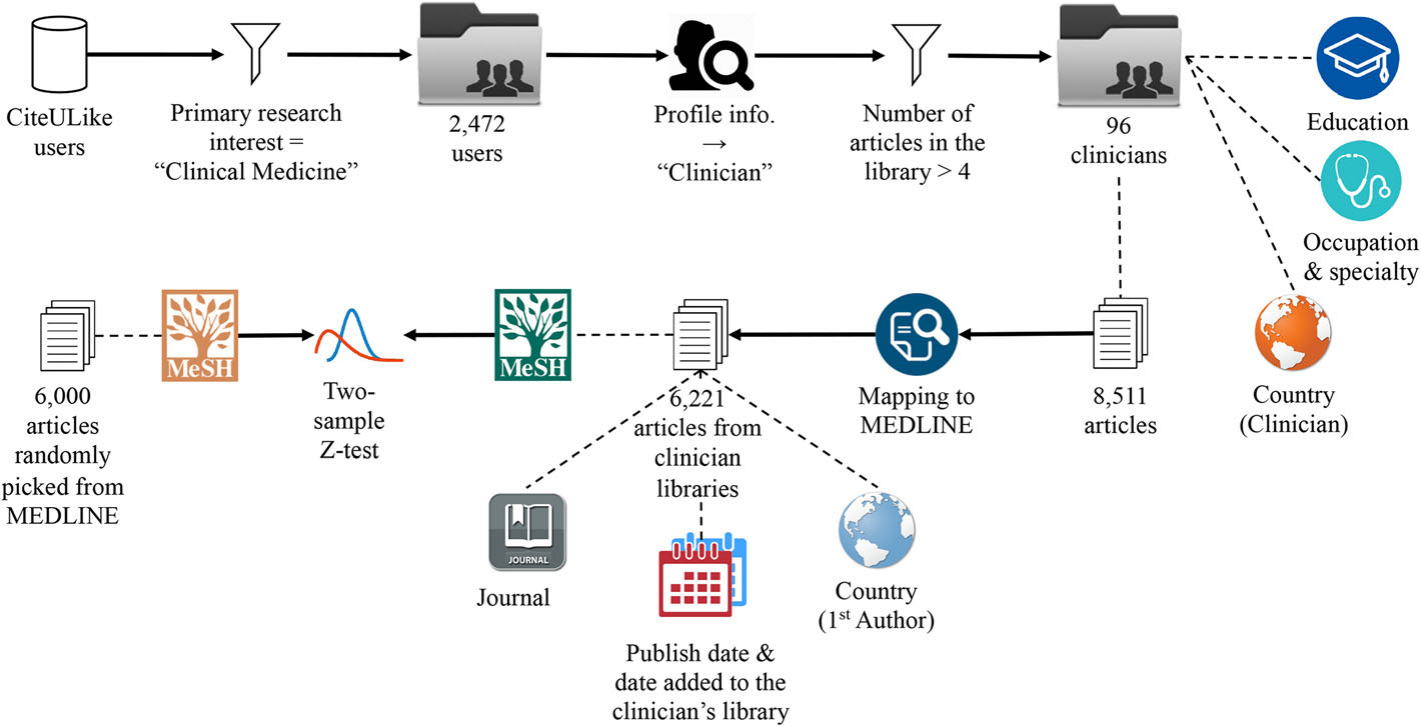
A workflow to determining types of research papers preferred by clinicians

The workflow of the entire study is illustrated in Fig. 1 with each step further described below.

### Data collection

We employed CiteULike.org [23] to identify a sample of clinicians who read scientific literature. Since 2004, CiteULike.org has provided free online reference management services with the goal of promoting the sharing of scientific references and fostering communication among researchers. It enables registered users to add publications they like to their own libraries and identify their research fields in profiles. The system then groups users of the same research field.

Moreover, the libraries and basic information of CiteULike.org users are openly shared on the website, making it an ideal data source choice for our study. We selected *clinical medicine* in the *primary research field* and retrieved 2,472 users on May 1, 2014. We manually verified whether each of these users is a clinician based on the combination of name, location, job title and affiliation information provided in the profile. In this process, we eliminated 91.7% of the users because significant amounts of information were missing from their profiles and we could not confirm that they were clinicians.

We further excluded 109 users who had fewer than five articles in their libraries because these users are less likely to be active users on CiteULike. Including them could complicate the analysis and results interpretation. After filtering according to these stringent selection criteria, our final sample of reading clinicians was comprised of 96 individuals, who claimed to be clinicians and had relatively complete information in their profiles. These 96 users cited 8,511 articles in their CiteULike libraries. For those articles with PubMed IDs (PMIDs), the unique identifier used by the PubMed search engine to access the MEDLINE bibliographic database of life sciences, we retrieved the complete abstracts in MEDLINE format. For the remainder, we searched PubMed using the article title and retrieved the publication if there was only one returned hit, which retrieved the exact match of the article most of the time, but not always. In this process, 2,290 articles (26.9%) could not be retrieved on account of missing bibliographic information, article exclusion from MEDLINE, or difficulty with the title search.

For example, some users did not list the complete article titles and other valid bibliographic information. Instead, they listed keywords identifiable only to themselves, such as *sharing and managing data*, *aging and the brain,* and *public health and Web 2.0*. These keywords were too general for PubMed to precisely locate corresponding articles. Other articles, such as “The Dos and Don’ts of PowerPoint Presentations” and “Inside Microsoft SQL Server 7.0 (Mps),” were not indexed by MEDLINE. Meanwhile, some articles were indexed in MEDLINE but could not be retrieved using the *title search* field in PubMed. We ultimately identified 6,221 publications that were cited in the user libraries, and we employed them in further analysis.

### Content analysis by MeSH

Medical Subject Headings (MeSH) is used by the National Library of Medicine (NLM) to annotate biomedical concepts and supporting facts addressed in each article indexed in the MEDLINE bibliographic database for information-retrieval purposes [24]. Each MEDLINE article is assigned two types of MeSH terms: major terms, which represent the main topics of the article, and minor terms, which represent the concepts and facts that are related to the experimental subjects and design attributes, such as humans or animals, adults or children, gender, and countries. MeSH annotations have been used in text mining and data mining tasks [25–27] and provided unique and key information on the topic and content preferences of the sampled clinicians in our study. All MeSH terms were included in the MEDLINE format abstracts that we downloaded.

We were eager to understand what content and topics, if different, that a dummy tool, without prior knowledge of its clinician users, might recommend. Therefore, we randomly selected 6,000 publications by sampling from PMIDs. We compared the MeSH terms from the real clinician reading libraries to the randomly sampled MEDLINE articles. The MeSH terms over-represented in the real clinician reading libraries were expected to suggest contents and topics that were more relevant to the clinicians. This information can provide useful clues for designing a personalized literature-recommender system that targets clinicians. In this experiment, we wrote Python scripts to extract and count the MeSH terms indexed in each article.

### Two-sample Z-test

The two-sample Z-test is often used for validating whether there is a significant difference between two groups based on a single categorical attribute. For example, it can validate whether there are more female vegetarians than male [28]. We chose this statistical test for our study because we intended to learn what contents and topics (in MeSH terms) are more interesting to clinicians. Our null hypothesis was that the frequency of MeSH term *t* in the clinician reading libraries was identical to that in the randomly sampled articles recommended by the dummy tool (*H*_*0*_: *f*_t,clinician_ = *f*_t,random_). The frequency was therein defined as the number of articles that were assigned the term *t* divided by the total number of articles in that group. We calculated the z-scores and two-side *p*-values in Excel to validate the null hypothesis.

### Multiple comparison correction

We considered the multiple comparison problem and avoided *p*-values that became ‘significant’ because of random effects [29] when performing Z-test for thousands of MeSH terms. We conducted the Benjamini-Hochberg false discovery rate (FDR) controlling procedure [30] in Excel to set more stringent *p*-value thresholds instead of 0.05.

## Results

**Fig. 2:**
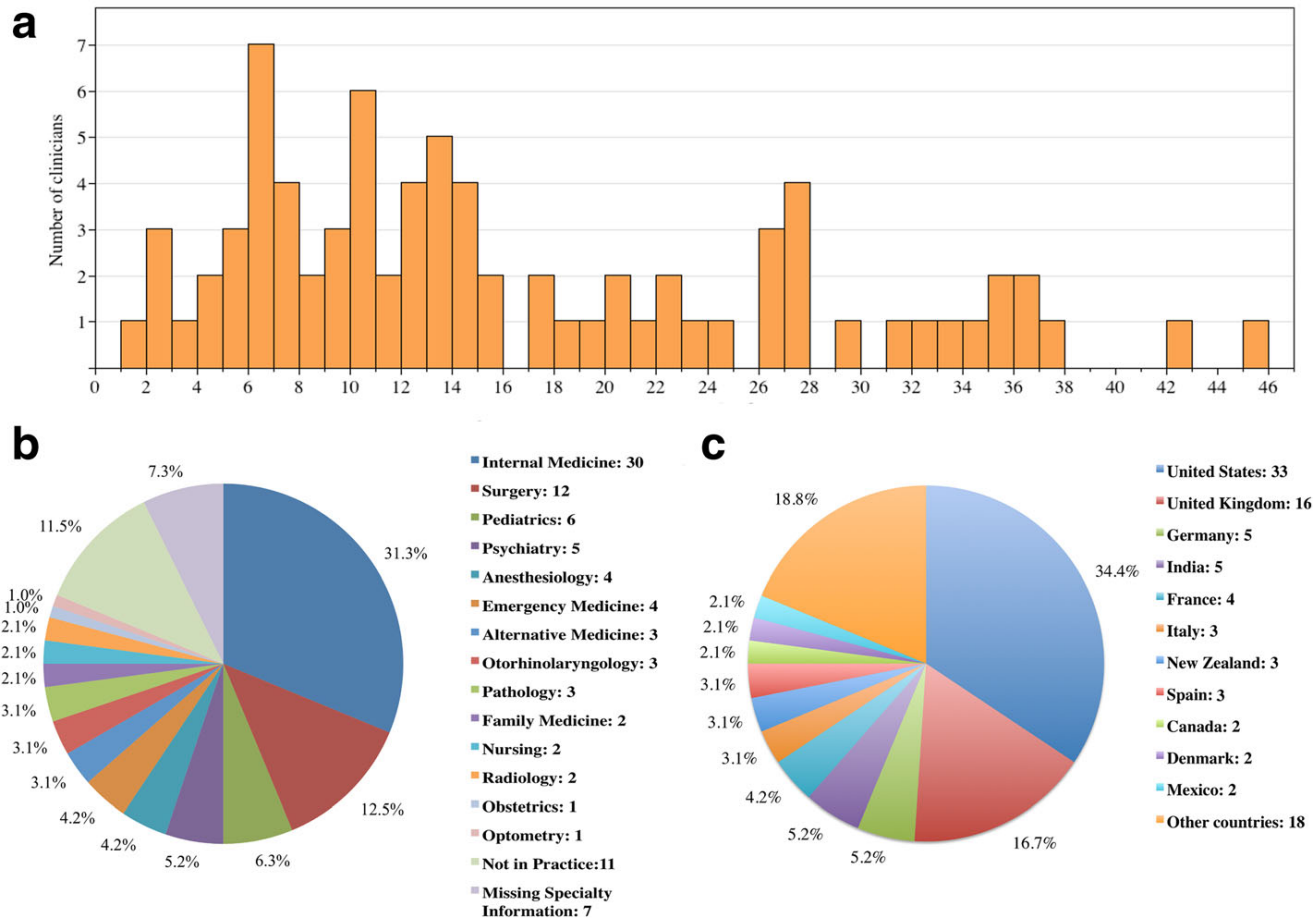
Demographic information for the sampled clinicians. **a** histogram of clinician practicing years after medical school graduation; **b** distribution of specialties; **c** distribution of countries of residence

We first examined the professional backgrounds of the 96 sampled clinicians. Of these clinicians, 58 were practicing doctors (60.4%), 19 were medical school faculty members (19.8%), 12 were medical doctors in atypical career paths, such as managerial/consulting positions in various healthcare organizations (12.5%), five were students advancing in post-graduate medical studies (5.2%), and two were practicing nurses (2.0%). In addition, we evaluated the time lengths of the clinicians’ medical practices. For each clinician, we calculated the number of years since he/she graduated from the professional school. The number of years range from 1 to 45, with an average of 16.0 (Fig. 2a).

In terms of specialty, 30 clinicians specialized in the internal medicine (31.3%), 12 in the surgery (12.5%), 6 in the pediatrics (6.3%), 5 in the psychiatry (5.2%), 25 in other medical specialty areas (26.0%, individual specialties with the number of clinicians in each were listed in Fig. 2b), 11 not actively seeing patients (11.5%) and 7 in an undisclosed specialty (7.3%). Geographically (Fig. 2c), 33 clinicians resided in the United States (34.4%), 16 in the United Kingdom (16.7%), five in the Germany (5.2%), five in the India (5.2%), four in the France (4.2%) and the remaining 23 clinicians were in other countries (24.0%, see Additional file 1: Table S1).

**Fig. 3:**
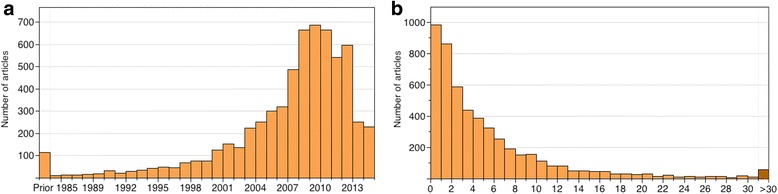
Temporal analysis of articles read by clinicians. **a** histogram of articles published each year; **b** histogram of age of articles when being read by clinicians (age = year read - publication year)

We then examined the clinician reading libraries. First, we plotted a histogram of the publication years for all 6,221 publications (Fig. 3a). Of the articles read by clinicians, 89.9% are published after 2000, with the peak centering between 2008 and 2010. In both 2013 and 2014, a significant decrease occurs. The 2013 decrease in the number of articles read by clinicians may be due to the fact that many users are no longer active on this website, whereas for 2014, we collected the data from CiteULike in May.

To examine how soon after an article is published that a clinician reads it, we plotted a histogram for the age of the article at the reading time, which is defined by the year when the article was read by a clinician minus its publication year. Figure 3b shows that articles published and read by clinicians in the same year are the highest, and a steady decreasing trend is evident when the article age increases. This result is strong evidence that clinicians in fact read the latest publications.

Interestingly, nine clinicians additionally read 51 papers published more than 30 years ago. These papers were published on journals with an average impact factor of 10.84 and have been cited for an average of 573.88 times according to Google Scholar, and thus can be considered as landmark articles in medical research. For example, “Studies of Illness in the Aged. Index of ADL: A Standardized Measure of Biological and Psychological Function” and “Functional Evaluation: The Barthel Index” were published in 1963 in the *Journal of the American Medical Association* (JAMA) and the *Maryland State Medical Journal*, with a Google Scholar citation of more than 7,000 and 9,400 times. These are the original publications of the most appropriate and extensively adopted measurement for evaluating functional status in the elderly population.

We summarized the types of publications for 6,221 articles and found that the majority are journal articles, including original research articles (3,698 or 59.4%), reviews (1,093 or 17.6%), reports of clinical trials (508 or 8.2%), case reports (259 or 4.2%), evaluation and validation studies (185 or 3.0%), comments (147 or 2.4%), and clinical guidelines (29 or 0.5%). The remaining 4.9% of articles belong to opinion and announcement categories, such as letters, editorials, and breaking news (see Additional file 1: Table S2).

In addition, we investigated what journals are read most often by clinicians and found that 6,221 articles are widely distributed among 1,664 journals. Nearly 50% (823) of the journals are cited only once in the clinician reading libraries, and 53 journals are cited 20 or more times (Table 1). Such a sparse distribution of journals suggests a need to further evaluate the impact of journals in a reliable recommender system model.

**Table 1:** Top journals read by the sampled clinicians and article count

*Arthroscopy: The Journal of Arthroscopic & Related Surgery*	88	*International Journal of Medical Informatics*	29
*Clinical Orthopaedics and Related Research*	84	*PLoS Medicine*	27
*British Medical Journal (Clinical Research Ed.)*	84	*Archives of Internal Medicine*	27
*New England Journal of Medicine*	78	*Annals of Internal Medicine*	27
*Journal of the American Medical Association*	76	*Spine*	26
*Science*	57	*BMC Medical Research Methodology*	26
*The Lancet*	57	*Cochrane Database of Systematic Reviews*	25
*Medical Education*	54	*Journal of Orthopaedic Research*	25
*BMC Medical Informatics and Decision Making*	54	*Anesthesia and Analgesia*	25
*Journal of Clinical Pathology*	53	*American Journal of Sports Medicine*	24
*PloS One*	50	*Studies in Health Technology and Informatics*	24
*Pain Medicine (Malden, Mass.)*	48	*Statistics in Medicine*	24
*Medical Teacher*	48	*Chest*	24
*Nature*	44	*American Journal of Roentgenology*	24
*Journal of Bone and Joint Surgery (American volume)*	42	*Journal of General Internal Medicine*	23
*Journal of the Association of American Medical Colleges*	39	*Biological Psychiatry*	23
*Journal of Bone and Joint Surgery (British volume)*	38	*Journal of Arthroplasty*	22
*Pediatrics*	37	*American Journal of Psychiatry*	22
*BMC Medical Education*	37	*Journal of Palliative Medicine*	22
*NeuroImage*	34	*Journal of Clinical Epidemiology*	22
*Journal of the American Medical Informatics Association*	33	*Journal of Biomedical Informatics*	22
*Proceedings of the National Academy of Sciences*	32	*Sociology of Health & Illness*	20
*Journal of Pain and Symptom Management*	32	*Radiology*	20
*Clinical Infectious Diseases*	32	*Orthopedics*	20
*Critical Care Medicine*	31	*Drug Safety: An International Journal of Medical Toxicology and Drug Experience*	20
*Social Science & Medicine*	29	*Critical Care (London, England)*	20
*Pain Physician*	29	Total: 1,933	

We later evaluated what journals that the clinicians of different specialty groups read (see Additional file 1: Table S3). The results indicate that prestige was not the most important factor when different specialty groups choose what scientific journals to read. Specialists tend to read journals closely related to their practice fields, rather than medical journals with high impact factors that target a broader readership. For instance, *Arthroscopy: The Journal of Arthroscopic & Related Surgery* is the most widely read journal among surgeons, while *The Lancet* ranks only in 108th place in their reading.

**Fig. 4:**
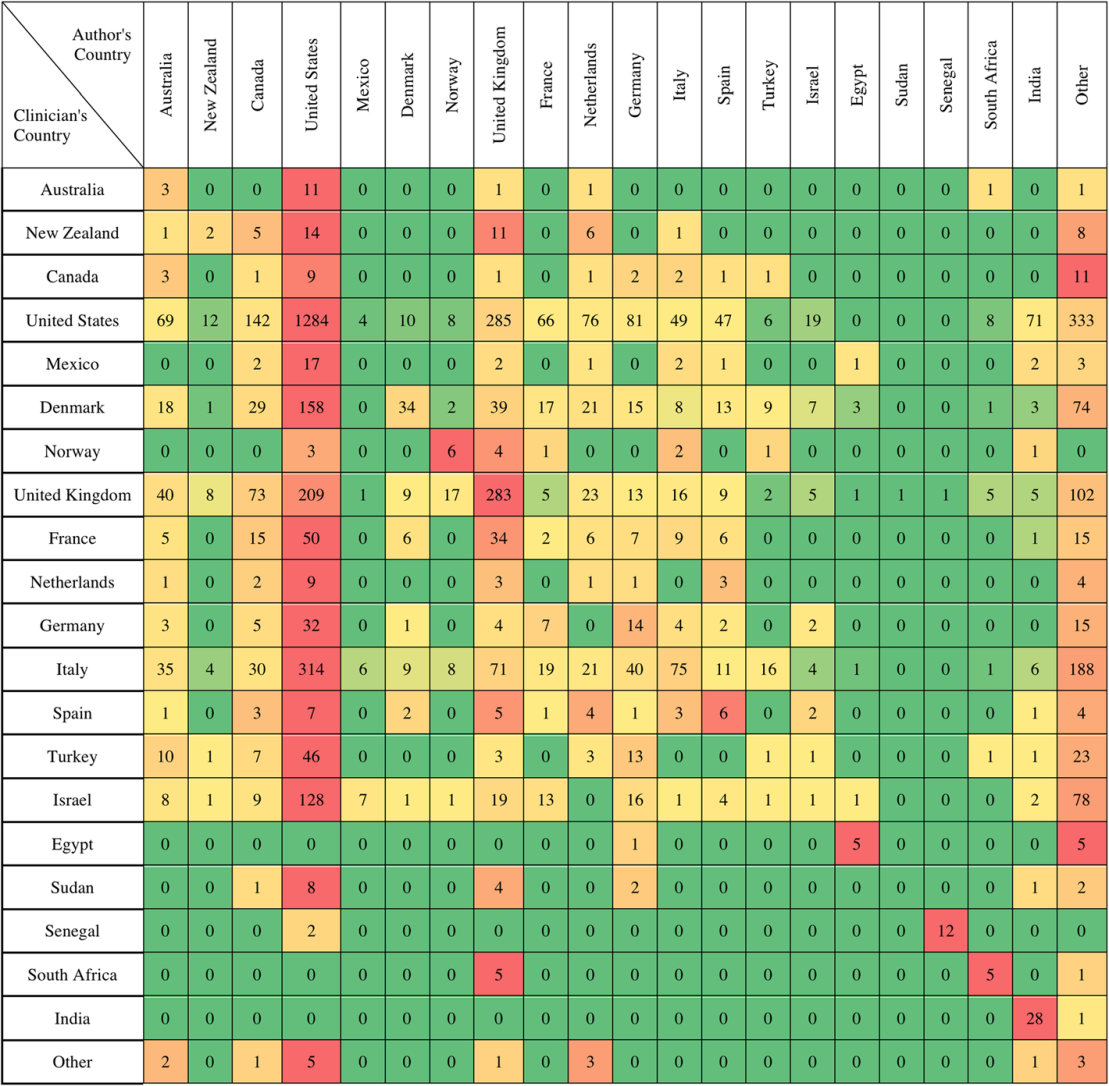
Clinician country of residence versus author country of residence in the reading libraries. Each row represents a country of residence of the sampled clinicians; each column represents the country of residence of the authors of the cited articles. The cell color changes from green (minimal count) to red (maximal count) for each row

To determine whether the country of residence and language or culture of practice can affect the clinician reading preferences, we analyzed the association between the clinician and author countries of residence for the articles in their libraries. If an article has authors from different countries, we used the first author’s country of residence. In the heat map of Fig. 4, each row represents a country of residence for the clinicians; each column represents the country of residence for the authors. The cell color changes horizontally from green (minimum number of articles) to red (maximum number of articles). According to the findings, articles written by American and British authors are extensively read by many clinicians in our sample, given the fact that these two countries publish a considerable amount of medical research. However, clinicians residing in Egypt, India, Norway, Senegal, and South Africa prefer works by authors of their own countries.

We performed a comprehensive statistical analysis to examine whether the topics of articles read by the sampled clinicians, in MeSH terms, differed from those recommended by the dummy tool without prior knowledge of the users. We found that 119 major MeSH terms and 288 minor MeSH terms have significantly different occurrence frequencies in the two groups (see Additional file 1: Table S4 and S5). Among the MeSH terms with the highest frequency variations in the two groups (Table 2), clear distinctions exist. Clinicians read more topics relating to patient issues and needs, such as pain, hip joints, drug therapy, surgery, arthroscopy, and therapeutic uses and adverse effects of analgesics. They prefer meta-analyses, reviews of literature, and quality of life research. Moreover, they are interested in research on human subjects, instead of molecule-, cell-, or animal-based studies, likely because human-based research is more relevant to treating their patients.

**Table 2:** MeSH term comparison between clinician reading libraries and a random sample

Major MeSH terms read more often by clinicians	Frequency: clinician	Frequency: random	*p*-value	diff	Major MeSH terms occurring more often in a random sample	Frequency: clinician	Frequency: random	*p*-value	diff
Pain/drug therapy	0.0186	0.0010	0.0000	0.0176	Polymorphism, Genetic	0.0003	0.0042	0.0000	−0.0038
Hip joint/surgery	0.0154	0.0000	0.0000	0.0154	DNA-Binding Proteins/metabolism	0.0002	0.0037	0.0000	−0.0035
Arthroscopy/methods	0.0130	0.0002	0.0000	0.0129	Models, Chemical	0.0002	0.0035	0.0000	−0.0033
Analgesics/opioids, therapeutic uses	0.0135	0.0007	0.0000	0.0128	Genetic Variation	0.0008	0.0035	0.0012	−0.0027
Meta-analysis as topic	0.0122	0.0000	0.0000	0.0122	Transcription Factors/metabolism	0.0005	0.0032	0.0005	−0.0027
Internet	0.0133	0.0013	0.0000	0.0120	Bacterial Proteins/metabolism	0.0000	0.0027	0.0000	−0.0027
Quality of life	0.0133	0.0032	0.0000	0.0102	Plant Extracts/pharmacology	0.0003	0.0025	0.0012	−0.0022
Physician–patient relationship	0.0116	0.0017	0.0000	0.0099	Enzyme Inhibitors/pharmacology	0.0002	0.0023	0.0006	−0.0022
Review literature as topic	0.0098	0.0002	0.0000	0.0096	DNA/chemistry	0.0000	0.0022	0.0002	−0.0022
Analgesics/opioids, adverse effects	0.0087	0.0000	0.0000	0.0087	Polymorphism, Genetic	0.0003	0.0042	0.0000	−0.0038
Minor MeSH terms read more often by clinicians	Frequency: clinician	Frequency: random	*p*-value	diff	Minor MeSH terms occurring more often in a random sample	Frequency: clinician	Frequency: random	*p*-value	diff
Humans	0.8608	0.6027	0.0000	0.2581	Animals	0.0754	0.2328	0.0000	−0.1574
Adult	0.2979	0.1625	0.0000	0.1354	Mice	0.0182	0.0675	0.0000	−0.0493
Female	0.4321	0.3025	0.0000	0.1296	Rats	0.0141	0.0458	0.0000	−0.0317
Male	0.4216	0.2937	0.0000	0.1280	Molecular sequence data	0.0035	0.0312	0.0000	−0.0276
Middle aged	0.2692	0.1490	0.0000	0.1202	Cell line	0.0031	0.0220	0.0000	−0.0189
Aged	0.1868	0.1040	0.0000	0.0828	Amino acid sequence	0.0014	0.0193	0.0000	−0.0179
Treatment outcome	0.0989	0.0515	0.0000	0.0474	Cells, cultured	0.0048	0.0217	0.0000	−0.0168
Adolescent	0.1100	0.0690	0.0000	0.0410	Base sequence	0.0031	0.0185	0.0000	−0.0154
Elderly: 80 and over	0.0722	0.0388	0.0000	0.0333	Cell line, tumor	0.0034	0.0170	0.0000	−0.0136
Prospective studies	0.0548	0.0240	0.0000	0.0308	Kinetics	0.0016	0.0152	0.0000	−0.0136

## Discussions

The proliferation of scientific publications and reduction of clinician reading times warrants the need for a method of enabling clinicians to quickly identify the latest results from scientific publications to more effectively practice evidence-based medicine. In the past decade, many clinicians have employed digital resources for the most recent and relevant findings and guidelines. Previous studies show that 60 to 70% of US clinicians access the Internet for professional purposes, and searching for literature in journals and databases is one of their most frequent online activities [31]. However, an overwhelming amount of information, coupled with the inadequate search skills of readers, remain major obstacles for clinicians to access research literature.

In recent years, several literature reading applications have been developed for clinicians to access research articles on smartphones and tablets [32], so that fragmented time between patient care could be better utilized. Some of them provided paper recommendation. For example, the Read by QxMD [33] suggests the latest research papers based on the users’ specialties and their choice of key words and journals. Docphin [34] tracks new and landmark papers related to the medical topics and authors specified by users. The mobile application offered by UpToDate.com [35] populates users’ reading list with articles picked by a board of medical experts. None of these implementations go beyond keyword-based recommendations and are not fully adopted by clinicians. A truly personalized literature-recommender system that alleviates the obstacles for clinician to access and read research literature requires more cognitive study to understand their reading preference and habits, which motivates us to carry out this work.

Our study advanced previous research [2–5, 7–9, 31, 36] by determining clinician reading preferences based on bibliographic and content aspects. We employed CiteULike, a contemporary data source, to identify the reading materials that are favored by the site’s clinician users. The publically available user profiles and reading library information on the website makes it more desirable for this study than other websites such as Mendeley. Moreover, compared to the widely used methods in previous studies [9] such as interviews, surveys, and tracking library access of limited samples within an institution, CiteULike offers two advantages. First, it is a non-invasive collection; the unnecessary response bias that is common in interview- or survey-based studies is avoided. Secondly, the sampled users on CiteULike represent clinicians from far more diverse geographic locations and medical specialties than in a specific hospital or institution.

In this study, we determined that research articles published in peer-reviewed journals are the most highly valued type; moreover, articles are usually read within the first 1 or 2 years after the publication date. Landmark papers in medical research history are also a significant category. Reviews, reports of clinical trials, meta-analysis studies, and case reports are likewise well represented across specialties and countries of practice.

In selection of the reading material, whether a paper is published in a prestigious journal with a high impact factor carries less weight than the topic of the paper and experimental design. The country of publication apparently also plays a role in reading preference. For example, some readers from Egypt, India, Norway, Senegal, and South Africa seem to prefer works by authors from their own countries (Fig. 4).

In content analysis, we determined that patient-oriented topics, meta-analyses, literature reviews, studies involving human subjects, and quality of life research are significantly more prevalent in clinician reading choices than in the overall publications.

These results provide important insights for designing a personalized literature recommender system that would be more welcome by clinicians, who are eager to learn the latest scientific discoveries relevant to patient care. For example, the system would possibly recommend not only latest research articles published in peer-reviewed journals, but also some landmark research works. The language and culture background, together with significantly over-represented MeSH terms, could be used in conjunction with the specialty information, so that recommendation could be optimized for different user groups.

However, the findings of this study must be considered in the context of the following limitations. First, the sample size of clinicians was small. The distribution of their demographic and professional attributes may not represent the entire clinician population. Secondly, we learned the paper reading preference of the sampled clinicians from articles cited in their CiteULike libraries. We assumed that the clinicians have read those articles in their libraries, which might not be true all the time. Thirdly, we randomly selected 6,000 papers from the MEDLINE bibliographic database based on PMID, and we used these papers to represent the possible recommendations by a dummy tool. This collection is not an ideal one for a comparison because it can include papers that clinicians may like to read. Consequently, we may have missed meaningful MeSH terms because the difference between groups was not statistically significant. In other words, we traded recall for precision when identifying the relevant MeSH terms. Finally, the content analysis was limited to user demographics and the bibliographic features documented by MEDLINE and CiteULike, such as practice specialty, publication year, and MeSH terms. On the other hand, analysis based on full-text articles is expected to provide a more comprehensive understanding of clinician preferences. Nevertheless, such a study would demand advanced text mining and natural language processing technologies.

## Conclusions

Despite the limitations of the present study, our findings on clinician reading preferences can serve as useful information for developing a personalized literature-recommender system for clinicians who work at the front-line of patient care. In the future, further research and development should be performed in this area so that clinicians can more effectively and conveniently access the most relevant scientific results. In addition, connecting clinicians and researchers for collaborations through a publication-based social network is another interesting aspect to be explored. Existing social network sites such as ResearchGate and Academia.edu have attracted a great number of scientists, but an online research community connecting researchers and clinicians is not available yet. Such a social network can improve the communication and collaboration between clinicians and medical scientists so that scientific breakthroughs can be applied to clinical settings faster, while medical scientists can more effectively learn and focus on patient-relevant problems.

## Additional file

Additional file 1: Table S1. Country of residence distributions of the clinicians. **Table S2.** Publication type distributions of the articles read by the clinicians. **Table S3.** Times of the journals read by the clinicians in each medical specialty group. **Table S4.** List of major MeSH terms having significant different frequencies between clinicians’ reading libraries and a random sample **Table S5.** List of minor MeSH terms having significant different frequencies between clinicians’ reading libraries and a random sample. (XLS 313 kb)

## Abbreviations

MeSH: Medical subject headings
NLM: National Library of Medicine
PMID: The unique identifier number for each article entered into PubMed System
